# *In vivo *versus *in vitro *protein abundance analysis of *Shigella dysenteriae *type 1 reveals changes in the expression of proteins involved in virulence, stress and energy metabolism

**DOI:** 10.1186/1471-2180-11-147

**Published:** 2011-06-24

**Authors:** Srilatha Kuntumalla, Quanshun Zhang, John C Braisted, Robert D Fleischmann, Scott N Peterson, Arthur Donohue-Rolfe, Saul Tzipori, Rembert Pieper

**Affiliations:** 1 Pathogen Functional Genomics Resource Center, J. Craig Venter Institute, 9704 Medical Center Drive, Rockville, MD 20850, USA; 2 Division of Infectious Diseases, Cummings School of Veterinary Medicine, Tufts University, North Grafton, MA 01536, USA

## Abstract

**Background:**

*Shigella dysenteriae *serotype 1 (SD1) causes the most severe form of epidemic bacillary dysentery. Quantitative proteome profiling of *Shigella dysenteriae *serotype 1 (SD1) *in vitro *(derived from LB cell cultures) and *in vivo *(derived from gnotobiotic piglets) was performed by 2D-LC-MS/MS and APEX, a label-free computationally modified spectral counting methodology.

**Results:**

Overall, 1761 proteins were quantitated at a 5% FDR (false discovery rate), including 1480 and 1505 from *in vitro *and *in vivo *samples, respectively. Identification of 350 cytoplasmic membrane and outer membrane (OM) proteins (38% of *in silico *predicted SD1 membrane proteome) contributed to the most extensive survey of the *Shigella *membrane proteome reported so far. Differential protein abundance analysis using statistical tests revealed that SD1 cells switched to an anaerobic energy metabolism under *in vivo *conditions, resulting in an increase in fermentative, propanoate, butanoate and nitrate metabolism. Abundance increases of transcription activators FNR and Nar supported the notion of a switch from aerobic to anaerobic respiration in the host gut environment. High *in vivo *abundances of proteins involved in acid resistance (GadB, AdiA) and mixed acid fermentation (PflA/PflB) indicated bacterial survival responses to acid stress, while increased abundance of oxidative stress proteins (YfiD/YfiF/SodB) implied that defense mechanisms against oxygen radicals were mobilized. Proteins involved in peptidoglycan turnover (MurB) were increased, while β-barrel OM proteins (OmpA), OM lipoproteins (NlpD), chaperones involved in OM protein folding pathways (YraP, NlpB) and lipopolysaccharide biosynthesis (Imp) were decreased, suggesting unexpected modulations of the outer membrane/peptidoglycan layers *in vivo*. Several virulence proteins of the Mxi-Spa type III secretion system and invasion plasmid antigens (Ipa proteins) required for invasion of colonic epithelial cells, and release of bacteria into the host cell cytosol were increased *in vivo*.

**Conclusions:**

Global proteomic profiling of SD1 comparing *in vivo vs. in vitro *proteomes revealed differential expression of proteins geared towards survival of the pathogen in the host gut environment, including increased abundance of proteins involved in anaerobic energy respiration, acid resistance and virulence. The immunogenic OspC2, OspC3 and IpgA virulence proteins were detected solely under *in vivo *conditions, lending credence to their candidacy as potential vaccine targets.

## Background

The Gram-negative bacterium *Shigella dysenteriae *serotype 1 (SD1) is among the most virulent serotypes of the four *Shigella *(*S*.) species (*S. dysenteriae*, *S. flexneri*, *S. sonnei *and *S. boydii*). SD1 is a causative agent of shigellosis, a severe form of epidemic bacillary dysentery in humans and primates [1,2]. Shigellosis is most prevalent in underdeveloped countries, with a mortality rate of 10-15% when untreated, killing about 1.1 million people of the roughly 120 million cases each year http://www.who.int/vaccine_research/diseases/diarrhoeal/en/index6.html. SD1 has an extremely low infectious dose of 10-100 organisms which has contributed to causing pandemic Shiga dysentery in several continents including Asia, Africa and Central America [2]. In addition to having a low infectious dose, multi-drug antibiotic resistance to more than six types of antibiotics (tetracycline, streptomycin, chloramphenicol, etc.) has developed in several *Shigella *serotypes [3]. *S. dysenteriae *is also very closely related to *Escherichia (E.) coli*, with certain strains of *E. coli *(Shiga toxin-producing *E. coli*, or STEC) producing the potent Shiga toxins (Stx) of which Stx1 is produced by SD1 as well [4]. Shiga toxin causes cell death primarily in the microvascular endothelium. A vaccine that is protective against *Shigella *serotypes is of utmost importance, and several attenuated vaccines are currently being developed and tested in human volunteers.

Components of the Type Three Secretion System (TTSS) encoded by a virulence plasmid are also involved in the pathogenesis of shigellosis [5]. Also called the Mxi-Spa system in *Shigella*, the TTSS is responsible for triggering entry into host epithelial cells and apoptosis in macrophages [6,7]. The TTSS is activated upon contact with host cells, leading to the integration of translocators in the host cell membranes which then promotes transit of effectors into host cells [8]. The TTSS and effector proteins thereby play an important role in infection and intra- and inter-cellular spreading of bacterial cells in the host intestinal epithelium [9]. O-antigens present in the cell surface lipopolysaccharide (LPS) of *Shigella *also contribute to its virulence [2]. The *Shigella *O-antigen comprises of a toxic lipid A moiety embedded in the bacterial outer membrane, a core sugar region and an exposed terminal O-polysaccharide. In SD1, the O-polysaccharide consists of tetrasaccharide repeats that contain repeat units of three rhamnose residues and one N-acetylglucosamine [2]. Enzymes essential for O-antigen biosynthesis are mostly encoded by chromosomal genes such as *galETKM *and *rfbBDACX *[10]. The terminal O-polysaccharide structures vary greatly among *Shigella*, thereby giving rise to the immunological specificity that has resulted in distinct serotypes. Although attenuated *Shigella *strains expressing genetically engineered O-antigens have been tested as vaccine candidates, an effective vaccine against *Shigella *remains elusive [2], possibly due to the diversity of the O-antigens.

Comprehensive proteomic profiling has the potential to identify novel virulence factors in *Shigella *that could form potential vaccine or therapeutic targets. Proteomic surveys of *Shigella *have mainly focused on *S. flexneri*, which causes endemic shigellosis. Extensive 2D-LC-MS/MS-based profiling of the *S. flexneri *membrane proteome by Wei *et al*. resulted in the identification of more than 600 *S. flexneri *proteins including *ca*. 200 integral and outer membrane proteins [11]. Immunoproteome analyses of *S. flexneri *identified several membrane proteins as being antigenic including OmpA, IpaD, Spa33, TolC and YaeT [12,13]. The *S. dysenteriae *strain Sd197 was the first *S. dysenteriae *genome to be sequenced [14], and included sequences of the chromosome, a large virulence-associated plasmid (pSD1_197) and a small plasmid (pSD197_Spa). This annotated SD1 genome was exploited in a comprehensive proteomic survey of *S. dysenteriae *strain Sd1617 *via *2D gel electrophoresis which resulted in the identification of 1061 distinct gene products [15]. Immunoproteome analysis of SD1 Sd1617 identified seven proteins including type III secretion system effectors OspC2 and IpaB as antigens. In this report, a quantitative global proteomic analysis of SD1 cells grown to stationary phase in culture (*in vitro*) *vs*. SD1 cells isolated from mammalian host environment (*in vivo*) was performed using 2D-LC-MS/MS and APEX, a label-free computationally modified spectral counting method [16]. Data from the SD1 *in vitro *and *in vivo *proteomes was analyzed for differential protein expression in the context of virulence and survival in the host.

## Methods

### Materials and reagents

All reagents for protein extraction from cell lysates and protein analysis by mass spectrometry (MS) were used as previously described [15,17]. RNase, DNase I (bovine pancreas), triethyl ammonium bicarbonate (TAB) buffer used for tryptic digestion, TCEP (Tris(2-carboxyethyl)phosphine) used as a reducing agent and the bicinchoninic acid (BCA) protein assay kit to estimate protein concentrations were purchased from Sigma-Aldrich (St. Louis, MO). The alkylating agent MMTS (methyl methanethiosulfonate) was purchased from Pierce (Rockford, IL). Sequencing grade porcine trypsin was obtained from Promega (Madison, WI). Triton X-100 was purchased from Calbiochem (LaJolla, CA). SDS-PAGE was performed according to instructions from Invitrogen.

### Bacterial strains and culture conditions

The strain Sd1617 of *Shigella dysenteriae *serotype 1 (SD1) strain was grown on tryptic soy agar plates (TSA) containing 0.05% Congo Red (w/v). SD1 *in vitro *samples were prepared by inoculating a single colony into Luria-Bertani (LB) medium grown to stationary phase at 37°C with agitation. The bacteria were harvested by centrifugation and washed twice with ice-cold PBS (6,000 × *g*, 15 min) at 4°C.

The inoculum for *in vivo *experiments was prepared by growing a typical SD1 colony selected from a TSA plate in LB medium overnight. Gnotobiotic piglets used for the animal experiments were delivered by Caesarian section at Tufts University Cummings School of Veterinary Medicine. Of several animals inoculated with SD1, three piglets were chosen for isolation of SD1 bacterial cells from the intestine in this comparative study. One of the piglets inoculated with 1 × 10^8 ^SD1 cells developed diarrhea 24 h later and was euthanized 4 d later when the gut contents were collected for bacterial purification. Another piglet inoculated with 5 × 10^8 ^SD1 cells developed diarrhea within 18 h and was euthanized 3 d post-inoculation. A third piglet inoculated with 5 × 10^9 ^SD1 cells developed diarrhea within 20 h and the gut contents collected 2 d post-inoculation. SD1 bacterial cells were isolated from the gut contents as described previously [15]. Briefly, the gut contents from cecum and colon were pooled and transferred to sterile histological cups placed on ice, suspended in ice-cold PBS at 4°C and pelleted at 5,000 × *g*. After resuspension of the pellet in 65% isotonic Percoll solution and centrifugation at 14,500 × *g*, the bacterial layer near the bottom was collected using a 3-5 ml syringe with needle. The bacteria were washed twice with ice-cold PBS at 4°C and processed for proteomic analysis.

### Lysis of *S. dysenteriae *cells and trypsin digestion of extracted proteins

After the PBS wash steps, bacterial cell pellets from *in vitro *or *in vivo *culture conditions were re-suspended in a hypotonic lysis buffer composed of 25 mM Tris-HCl (pH 7.8) with 150 μg/mL lysozyme, 0.05% Triton X-100, 5 mM EDTA, protease inhibitors (1 mM benzamidine and AEBSF) for 30 min at room temperature (RT) with gentle agitation. The samples were then placed at -80°C until further processing. For nucleic acid digestion, bacterial samples suspended in the lysis buffer were thawed and gently agitated for 1 h at RT after the addition of DNase I, RNase and leupeptin (10 μg/mL each) and 20 mM MgCl_2_. Cell lysates were centrifuged at 16,000 × *g *for 30 min at 4°C, and the supernatants containing bacterial cell lysate proteins were recovered.

Following cell lysis, the extracted bacterial proteins were precipitated in six volumes of ice-cold acetone at -20°C for at least 1 h. Acetone-precipitated proteins were recovered as a pellet after centrifugation at 5,000 × *g *for 10 min. The protein pellet was resuspended in 0.1 M TAB buffer, pH 8.5, and the total protein concentration measured using the BCA assay. Proteins were denatured in 0.1% SDS and reduced using 5 mM TCEP for 1 h at 37°C, followed by alkylation using 10 mM MMTS for 1 h at RT [18]. In-solution trypsin digestion of the complex protein mixture was performed by the addition of trypsin at 1:25 for 5 h at 37°C followed by 1:50 digestion overnight. The tryptic digested samples were applied to SDS-PAGE to check for extensive digestion.

### Mass spectrometry analysis of tryptic peptides

Methods for mass spectrometry (MS) analysis were previously described in detail [17]. Briefly, tryptic peptide digests (*ca*. 100 μg) were fractionated by 2D-LC-MS/MS, first using a Polysulfoethyl-A SCX column (4.6 × 50 mm, Nest Group, USA) followed by an Agilent 1100 series solvent delivery system (Agilent, Palo Alto, CA) online with a nano-electrospray LC-MS/MS system (LTQ-IT mass spectrometer, Thermo-Finnigan, San Jose, CA). SCX fractions were delivered from 96-well plates onto a PicoTip microcapillary reversed-phase column (BioBasic C_18_, 75 μm × 10 cm, New Objective, Woburn, MA) at a flow rate of 350 nL/min. Spectra were acquired in automated MS/MS mode with the top five parent ions selected for fragmentation using collision energy of 35%. LC-MS/MS was performed in three sequential *m/z *subscans (300-650, 650-900, 900-1500 *m/z*) to increase the sampling depth [16].

MS and MS/MS data from sequential runs were combined for search against the latest release of the *S. dysenteriae *Sd197 genome database in NCBInr using the Mascot search engine *v*.2.2 (Matrix Science, London, UK). This database contained 4502 protein sequences, including 231 proteins encoded by the two SD1 plasmids. Mascot search parameters allowed for tryptic specificity of up to one missed cleavage, with methylthio-modifications of cysteine as a fixed modification and oxidation of methionine as a variable modification. The LTQ search parameters for +1, +2 and +3 ions included mass error tolerances of ± 1.4 Da for peptide ions and ± 0.5 Da for fragment ions. The false discovery rate (FDR) for peptide identifications was determined using the Mascot decoy database search option, with searches against a randomized *S. dysenteriae *Sd197 protein decoy database. Mascot search results of replicate 2D-LC-MS/MS experiments were further validated by estimating the FDR [19]*via *PeptideProphet™ and ProteinProphet™ [20] which are part of the Trans-Proteomic Pipeline (TPP) available at http://tools.proteomecenter.org/wiki/index.php?title=Software:TPP.

### APEX quantitation of SD1 cell lysate LC-MS/MS datasets

APEX quantitation of SD1 proteins was performed using the APEX Quantitative Proteomics Tool [21]*v*.1.1 as described previously [17]. Briefly, three steps were performed, building a SD1 training dataset, computing SD1 protein *O_i _*(expected number of unique proteotypic peptides for protein *i*) values, and calculating SD1 protein APEX abundances. Proteins in the training dataset were comprised of the 100 most abundant SD1 proteins based on high spectral counts per protein and high protein and peptide identification probabilities [22]. The training dataset. ARFF file was constructed based on 35 peptide physicochemical properties deemed significant for the computational prediction of proteotypic peptides [16,23]. To compute SD1 protein *O_i _*values, the Random Forest classifier algorithm was applied to the SD1 training dataset constructed in the previous step, and then to all tryptic peptides generated *in silico *from the SD1 proteome to enable computation of SD1 protein *O_i _*values. APEX abundances of the SD1 proteins observed by 2D-LC-MS/MS were calculated using the protXML files generated from the PeptideProphet™ and ProteinProphet™ validation of the Mascot search results and the SD1 protein *O_i _*values. While data from the technical replicates (three to five) for each of the three biological samples were pooled in the analysis, data from the biological replicates were analyzed separately under *in vitro *and *in vivo *conditions. A <5% FDR was chosen, along with a normalization factor of 2.5 × 10^6^. The normalization factor in the APEX tool is equivalent to the term *C *in the APEX equation [16], which represents the total concentration of protein molecules per cell. Since *S. dysenteriae *is closely related to *E. coli*, the total number of protein molecules/cell estimated at 2-3 × 10^6 ^for *E. coli *[16] was used as a normalization factor in the APEX abundance measurements of *S. dysenteriae *proteins.

### Bioinformatic analysis tools

*In silico *predictions of subcellular protein localizations were obtained using PSORTb *v*.2.0 searches [24] of the *S. dysenteriae *Sd197 proteins. In cases where the PSORTb analysis was inconclusive, the datasets were queried by five other algorithms (SignalP [25], TatP [26], TMHMM [27], BOMP [28] and LipoP [29]) to predict motifs for export signal sequences, TMD proteins and lipoproteins in SD1 proteins.

### Statistical analysis, clustering and pathway analysis of SD1 proteomic datasets

Differential protein expression analysis of the *in vitro vs. in vivo *proteomes was examined using a two-tailed Z-test [16] incorporated into the APEX tool [21]. The p-values from the Z-test obtained for the proteins common to the *in vitro *and *in vivo *samples were subjected to the Benjamini-Hochberg (B-H) multiple test correction available from the open source R statistical package http://www.r-project.org to estimate the false discovery rate (FDR). Further statistical analysis and clustering of the data were performed using the MeV *v*.4.4 (Multiexperiment Viewer) software tool, an application designed for detailed statistical analysis of large-scale quantitative datasets [30,31]. A two-class SAM (Significance Analysis for Microarrays) was performed, and a heat map generated by clustering the data using HCL (Hierarchial Clustering) and Euclidean distance in MeV. To determine the reproducibility of the datasets, a pairwise Pearson's correlation plot was constructed to correlate protein abundance values obtained for each protein from replicate analyses. For pathway analysis, the *S. dysenteriae *Sd197 KEGG pathway was obtained from the KEGG database [32]. The KEGG pathway was loaded into Katsura *v*.1.0 (JCVI), which is an open source software application for exploring the KEGG metabolic pathway coverage and expression available at http://pfgrc.jcvi.org/index.php/bioinformatics/katsura.html. To identify the SD1 metabolic pathways and functional proteins that were altered under *in vivo *conditions as compared to *in vitro *conditions, each pathway was examined for proteins exhibiting higher or lower protein abundance values based on the two-tailed Z-test analysis.

## Results and Discussion

### Global profiling of *S. dysenteriae *strain Sd1617 *in vitro *and *in vivo *proteomes

*Shigella dysenteriae *serotype 1 (SD1), which possesses the cytotoxic Shiga toxin (Stx), causes deadly epidemics in many poor countries [14]. However, no effective vaccine for this pathogenic organism is currently available although there are several attenuated strains at different stages of development [2]. Proteomic analysis of *S. dysenteriae *is a strategy to identify novel vaccine and therapeutic drug targets. A gnotobiotic piglet model was recently developed [33] to serve as an alternative to a primate model to study infections with the highly host-specific pathogen *S. dysenteriae *[15,34]. SD1 bacterial cells were collected from stationary phase suspension cultures in LB broth (referred to as '*in vitro'*) and from the gut of several infected gnotobiotic piglets (referred to as '*in vivo*'). The lack of microflora in gnotobiotic animals and the ability to recover more than 10^9 ^purified SD1 bacteria from *in vivo *conditions allowed unique studies of the nature of the pathogen's direct interaction with the host tissue in the absence of other interfering microflora.

**Figure 1:**
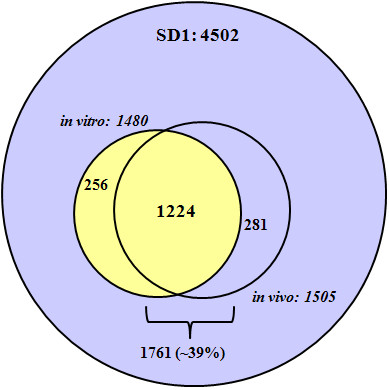
**Euler/Venn diagram representations of *S. dysenteriae *serotype 1 (SD1) proteins**. Of the 4502 proteins predicted for the SD1 genome, 1761 proteins were identified at a 5% false discovery rate (FDR), with 1480 proteins identified from the *in vitro *analysis, and 1505 proteins from the *in vivo *analysis.

A preliminary 2D gel-based survey of the SD1 proteome from the piglet intestinal environment was reported previously [15]. Here, the scope of the differential proteomic analysis was expanded using three to five technical and three biological replicates from both *in vitro *and *in vivo *groups. We resorted to a strategy combining the benefits of 2D-LC-MS/MS for a comprehensive coverage of proteins, and APEX (a modified spectral counting method for protein expression measurements derived from LC-MS/MS datasets). The *in vitro *analysis resulted in the identification of 1480 proteins while the *in vivo *analysis identified 1505 proteins at a 5% false discovery rate (FDR). 1224 proteins were common to both samples, with 256 and 281 proteins unique to the *in vitro *and *in vivo *analyses, respectively (Figure 1). Genome sequencing of the strain Sd197 suggested 4271 chromosomal ORFs, 223 plasmid pSD1_197-encoded ORFs and 8 plasmid pSD197_spA-encoded ORFs [14]. Combining LC-MS/MS data from all experiments and assuming a 5% FDR, 1761 proteins comprising 39% of the SD1 proteome were identified across a wide M_r _(4.3 - 176.5 kDa) and pI (3.59 - 11.84) range (Additional File 1, Table S1). 105 and 95 proteins were identified based on a single peptide match from the *in vitro *and *in vivo *analyses, respectively. Since small or low abundance proteins are frequently identified by one or two peptides [19], validation of the single peptide match proteins was performed by validating the spectrum manually. Of the 231 proteins encoded by the two plasmids pSD1_197 and pSD197_spA, 66 and 3 proteins were identified, respectively. This included 15 Mxi-Spa proteins and 16 effectors/chaperones of the type III secretion system (TTSS) clustered in the *ipa *gene locus of pSD1_197. Wei *et al*. [11] identified 45 of the orthologous *S. flexneri *proteins expressed from the plasmid pCP301, including 8 Mxi-Spa proteins and 11 effectors/chaperones. The comparison supports the notion that expression of these genes is important in the proper functioning of the TTSS of both *Shigella *species.

**Figure 2:**
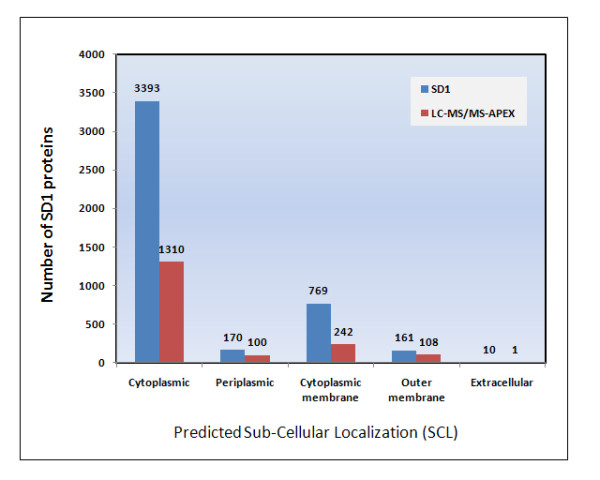
**Sub-cellular localization (SCL) of SD1 proteins**. SCL of 4502 proteins encoded by the SD1 genome was predicted using the bioinformatic algorithms PSORTb, SignalP, TatP, TMHMM, BOMP, LipoP and KEGG. 350 outer and inner membrane proteins corresponding to *ca*. 38% of the SD1 membrane proteome, and 1410 cytoplasmic and periplasmic proteins representing *ca*. 39% of SD1 soluble proteins were identified.

Subcellular localizations (SCL) of all 1761 identified SD1 proteins were determined, either based on *in silico *predictions by the tool PSORTb or by the combination of short motifs recognized in protein sequences by six different algorithms (SignalP, TatP, TMHMM, BOMP, LipoP and KEGG pathway role). Data from the latter categorization are displayed in Figure 2, with most proteins (1310) being assigned to the cytoplasm. As membrane proteins are often of particular interest in the context of virulence, they were also selectively surveyed in a study on *S. flexneri *2a [11], yielding approximately 35 outer membrane (OM) and 159 integral cytoplasmic membrane (CM) proteins. SCL prediction of our data yielded 350 membrane proteins (including 108 OM and 242 CM proteins), contributing to an extensive survey of the *Shigella *membrane proteome. Many peripheral, integral and lipid-anchored membrane proteins could also be quantitated applying the APEX tool. This is a marked advantage of 2D-LC-MS/MS over 2D gel-based proteomic surveys. For example, we were able to obtain quantitative estimates for numerous membrane proteins, some of them part of complexes. This included 7 of the 8 F0F1 ATP synthase subunits predicted for SD1 http://biocyc.org, 11 of the 13 NADH dehydrogenase (Nuo) subunits, all three formate dehydrogenase subunits (FdoG/H/I), all four cytochrome oxidase subunits (CydA/B/C/D), β-barrel OM porins (OmpA, OmpC, OmpX), multidrug efflux transporters (MdlA, MdlB, YdhE, YhiU, EmrA, EmrY) and 15 structural components of the bacterial Mxi_Spa apparatus. Most proteins or their orthologs which were described as being immunogenic by Ying *et al*. [12,35] in *S. flexneri *and Pieper *et al*. in *S. dysenteriae *(15), were also identified in this SD1 dataset (OmpA, YaeT, OppA, DnaK, ClpB, Pgm, AtpA, AtpD, LpdA, Gnd, Tst, MglB, FusA, ManX, TolC, UshA, OspC2, VirB and IpaB).

Highly abundant SD1 proteins, *in vivo *and *in vitro*, were implicated in energy/carbon metabolism and protein synthesis. This included glycolytic enzymes (PckA, GapA, Tpi, Fba, Pgk, GpmA, Eno), elongation factors (FusA, TufA, Tsf), several ribosomal protein subunits (RpsD/K/M, RplC/D/E, RpmC/D/J), and stress response proteins (WrbA, AhpC, SodB). Proteins with global regulatory functions in the cellular stress response were identified *in vivo *as well as *in vitro *(Hns, RpoS and CpxR). In summary, SD1 cells produced proteins essential for growth and cell integrity (energy generation, protein synthesis, cell envelope structure) as well as response to cellular and environmental stresses in high abundance.

### Differential abundance analyses of the SD1 *in vitro *and *in vivo *proteomes

**Figure 3:**
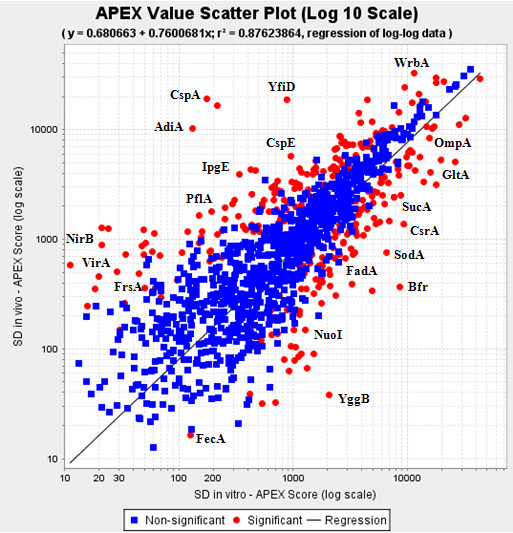
**SD1 differential protein expression analysis using the two-tailed Z-test**. Approximately 300 proteins were found to be differentially expressed at 99% confidence, including 151 in vivo and 142 in vitro SD1 proteins using the two-tailed Z-test utility in the APEX tool application.

**Figure 4:**
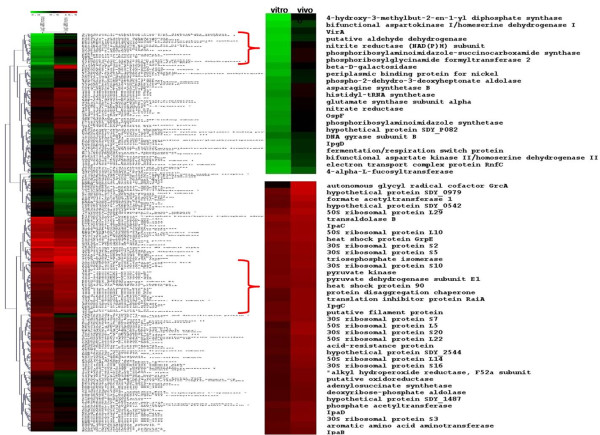
**Hierarchial clustering (HCL) analysis of differentially expressed SD1 proteins based on APEX abundance values using MeV**. Protein abundance values from the *in vitro *sample are represented on the left, with *in vivo *protein abundances on the right. Abundance magnitude is depicted as a color gradient, with red indicating an increase in protein abundance, green indicating a corresponding decrease in abundance, and black for the median level of abundance. Based on biological interests, example clusters are enlarged to depict differentially expressed proteins.

**Figure 5:**
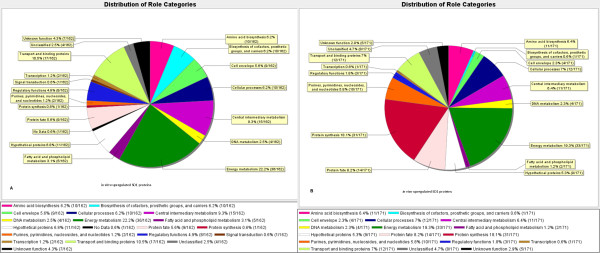
**Representation of functional role categories of SD1 proteins**. Proteins identified from 2D-LC-MS/MS experiments of *S. dysenteria*e cells were analyzed based on protein functional assignments in the CMR database for the genome of SD1 strain Sd197. Distribution of role categories of SD1 proteins cultured from stationary phase cells (*in vitro*) are shown in the panel on the left (5A) and cells isolated from gut environment of infected piglets (*in vivo*) are depicted on the right (5B).

Data from three biological replicates pertaining to *in vivo *and *in vitro *conditions were subjected to statistical analyses. The biological replicate analyses were pooled for the Z-test, and analyzed separately by the SAM test. Differential expression analysis of the *in vitro vs. in vivo *proteomes using a two-tailed Z-test resulted in *ca*. 300 proteins identified as being differentially abundant at a 99% confidence level (Figure 3), while the SAM test identified *ca*. 90 differentially expressed proteins (Additional File 2, Table S2). As the SAM test takes into account the biological variability between replicates, it is more conservative at estimating the differential protein expression given the dynamic range of the biological data which may inflate variance measures. The Benjamini-Hochberg (B-H) multiple test correction performed on the 1224 proteins common to the *in vitro *and *in vivo *samples estimated the FDR at <5% for the *ca*. 300 differentially expressed proteins identified from the Z-test (Additional Files 1 and 2, Tables S1 and S2). Hierarchial clustering of the data resulted in several major clusters of similarly expressed proteins (Figure 4). Selection of two clusters magnified in Figure 4 was based on biological interest in the set of proteins that exhibited differential abundance values. For example, one of the clusters harbored numerous ribosomal proteins and several Ipa/Ipg host cell invasion proteins, all of which were clearly increased in abundance *in vivo*. Another cluster harbored several enzymes indicative of the shift from aerobic to anaerobic energy generation. Protein functional role categories of the differentially expressed proteins were assigned according to the CMR database http://cmr.jcvi.org and are displayed in Figure 5. Several cellular processes appeared to be activated in the intestinal environment comparing the percentage of abundance-changed proteins per role category in both the *in vitro *(Figure 5a) and the *in vivo *(Figure 5b) groups. Most striking were the changes in protein synthesis (0.6% *vs*. 18.1% *in vitro *and *in vivo*, respectively) and purine, pyrimidine and nucleotide biosynthesis (1.2% *vs*. 5.8%). In contrast, activity decreases *in vivo *were denoted for regulatory processes (4.9% *vs*. 1.8%), cell envelope functions (5.6% *vs*. 2.3%) and transport (10.5% *vs*. 7%). Overall, the graphic in Figure 5 clearly illustrates that the SD1 cells adapt to the host intestinal environment by alternating a multitude of their cellular pathways and processes.

Differential expression analysis of the APEX datasets revealed several biochemical processes that appeared to be important for the pathogen to infect the piglets and to survive in their intestinal environment. Strongly altered abundances in the *in vivo *environment pertained to proteins involved in mechanisms of acid resistance (GadB, AdiA, HdeB, WrbA), the switch from aerobic to anaerobic respiration and mixed acid fermentation (PflA, PflB, PykF, Pta), oxidative stress (YfiD, YfiF, SodB) and other general cellular stress responses involving cold and heat shock proteins (CspA, CspE, ClpB). The *in vivo *responses suggested enhanced bacterial stress under oxygen- and nutrient-limited conditions in the host gut environment. In contrast, the *in vitro *proteome was defined by high abundances of enzymes involved in fatty acid oxidation (FadA, FadB, FadD, etc.) and aerobic respiration (GltA, IcdA, SdhA, SucA, etc.). Several proteins linked to the type III secretion apparatus were increased in abundance, or found solely under *in vivo *conditions including chaperones for effector proteins (IpgA/B/C/D/E), transcription activators (VirB), translocators (IpaA/B/C/D) and effectors (IcsB, VirA, OspF).

### Transition from aerobic to anaerobic respiration and fermentation in SD1 bacterial cells *in vivo*

The pathogenic *S. dysenteriae *is a facultative anaerobe which can switch to an anaerobic energy metabolism when starved of oxygen in the host large intestine. Proteins involved in energy metabolism formed the largest category of abundance-changed proteins under both *in vitro *and *in vivo *conditions (Figure 5), indicative of the impact of the intestinal environment on the SD1 cell's energy generation pathways. We have previously reported in a 2D gel-based proteomic analysis that the intestinal environment resulted in a shift from aerobic respiration to fermentation in SD1 cells (15). A more comprehensive dataset was obtained in this study and highlights the advantages of 2D-LC-MS/MS and APEX over differential 2D gel display. The former approach is not only more sensitive, as it strongly increased the number of profiled low abundance proteins, but also revealed marked advantages *via *the quantitation of hydrophobic CM and OM proteins.

It was confirmed that most of the tricarboxylic acid (TCA) cycle enzymes were strongly decreased in SD1 cells *in vivo*, such as GltA, IcdA, SucA, SucB, SucC, SucD, SdhA, SdhB and Mdh. The abundance changes of these and following enzymes are provided in Additional File 1, Table S1. The TCA cycle was clearly less active under anaerobic (*in vivo*) than aerobic/microaerophilic (*in vitro*) conditions. New evidence was obtained that the major enzyme complex contributing electron donors in the aerobic respiratory chain, NADH:ubiquinone dehydrogenase I, was markedly less active *in vivo*. Nearly all of the subunits (NuoA/B/C/E/F/G/H/I/K/L/N) were decreased in abundance *in vivo*. Likewise, a second major electron donor enzyme complex known to be active under aerobic conditions in *E. coli*, succinate dehydrogenase, featured strong decreases *in vivo *(SdhA/B/C/D). The cytochrome b562 protein CybC was also strongly decreased *in vivo*.

The question arose as to which electron donor complexes substituted for Sdh and Nuo *in vivo *to support anaerobic and microaerobic respiration. Surprisingly, subunits of formate dehydrogenase (FdoG/H/I) were moderately decreased in abundance *in vivo*, whereas Fdn was not detected at all. Fdn is purportedly a selective electron donor for anaerobic respitration http://ecocyc.org. FNR (fumarate and nitrate reductase regulator) and NarP, both components of the complex regulatory system of respiratory enzymes, were increased in abundance in SD1 cells *in vivo*. FNR also activates sRNAs that degrade mRNAs coding for proteins involved in aerobic respiration [36]. This data supported the notion that major metabolic shifts in aerobic/anaerobic respiration were regulated at the post-transcriptional level. Periplasmic nitrate reductase, the Nap complex, was strongly increased *in vivo *upon comparing the abundances of the subunits NapA, NapB and NapC. In *E. coli*, Nap was shown to be induced under anaerobic conditions and also regulated by FNR and NarP [37]. Nap appears to act as an electron acceptor under low nitrate conditions in *E. coli*, suggesting a similar function in SD1. The nitrite reductase (NirB/NirD) was also increased *in vivo*. This complex has been associated with nitrite detoxification and appears to be metabolically linked to the activity of the periplasmic Nap protein.

Low abundance of electron donors of respiratory complexes was indicative of a switch to mixed acid fermentation *in vivo*. Indeed, proteomic evidence strongly supported the assumption that mixed acid fermentation and substrate level phosphorylation substituted for the low abundance of electron donors. Dramatic increases were noted for subunits of pyruvate formate lyase complexes. This included the activating enzyme PflA, formate acetyltransferases (PflB, TdcE), a putative formate acetyltransferase YbiW, and the stress-induced alternate pyruvate formate lyase YfiD. Other mixed acid fermentation branches also appeared to be more active *in vivo*, such as the one initiated by PykA/PykF, which is coupled to acetate secretion *via *the phosphate acetyltransferase (Pta) and acetate kinase (AckA) activities. Interestingly, the fermentation/respiration switch protein FrsA was increased in abundance *in vivo*. In summary, this data provided comprehensive molecular evidence for the shift from aerobic/microaerobic respiration to fermentation in SD1 cells in the host intestinal environment.

Fermentation pathways and associated stress responses have been characterized in *E. coli *[38]. The dramatic quantitative increase of YfiD is indicative of the fact that the glycyl radical protein is a key enzyme required to maintain the activity of PflA/PflB. YfiD has also been linked to low pH stress; the notion that this protein is essential for the survival of *Shigella *in the host gastrointestinal environment is intriguing, and makes YfiD a prospective drug target. The *E. coli *YfiD was also reported to be induced under acidic conditions *in vitro *[39]. The stress-induced alternate pyruvate formate-lyase YfiD appears to replace PflB upon oxidative inactivation during oxidative stress conditions in *E. coli *[40], thus supporting a critical metabolic role of the pyruvate-formate lyase PflA/YfiD in SD1 cells *in vivo*. Other mixed acid fermentation branches operating *in vivo *included reductive pathways for lactate and ethanol, each generating NAD+ from NADH. In summary, survey of proteomic data supports strong activity increases in mixed acid fermentation, whereas the TCA cycle and aerobic processes were decreased correspondingly in SD1 cells localized in the anaerobic piglet intestine environment.

### Response of SD1 cells *in vivo *to acid stress and toxic conditions

Acid resistance systems enable the survival of commensal and pathogenic *E. coli *strains in the acidic stomach and host intestinal environment, and neutralize intracellular acidic fermentation products [41,42]. Strong abundance changes of several SD1 enzymes contributing to pH homeostasis in this pathogen were identified in a recent study (15). This data lends further credence to the important function of two acid resistance (AR) systems, AdiA/AdiC and GadB/GadC, to maintain the intracellular pH in SD1 cells during gastric passage and, possibly, as a result of increased generation of acidic fermentation products in the intestine. The orthologous AR systems were previously characterized in *E. coli *[42]. While increases of AdiA were strong *in vivo*, they also revealed variability comparing the piglet-derived samples (Additional File 3, Table S3). Of the transcription factors GadX, EvgA and YdeO, all reported to influence expression of acid resistance genes [42,43], EvgA was increased *in vivo *suggesting a key regulatory role of EvgA during acidic stress in SD1. As also reported earlier (15), two periplasmic acid resistance chaperones (HdeA, HdeB) which protect periplasmic proteins from aggregation and denaturation at low pH were increased in SD1 cells *in vivo*. Hde proteins expose hydrophobic protein surfaces at low pH and initiate formation of aggregates with denatured periplasmic protein substrates [44,45].

Host invasion by SD1 implicates the invasion and release from gut epithelial cells and cells of the innate and adaptive immune systems. SD1 cells are exposed to toxic molecules produced and secreted by cells of the immune system. We mined proteomic data for indicators of the molecular response to toxins. The most intriguing finding was the high abundance of nitric oxide (NO) dioxygenase (HmpA) detected only under *in vivo *conditions. NO is known to be produced in large quantities in macrophages and is toxic to intracellular bacteria. In *M. tuberculosis*, the nitric oxide dioxygenase HbN was shown to be important for nitrite detoxification [46]. A hydroxylamine reductase, YbjW, also scavenges toxic by-products of nitrogen metabolism and was detected only *in vivo *in SD1 cells. We speculate that the expression of these SD1 enzymes reflected memory of a previous intracellular life stage. Both enzymes are interesting targets for inhibitory drug design. To cope with oxidative stress, SD1 cells displayed increased abundances of superoxide dismutases (SOD) whose expression has been linked to oxygen availability in *E. coli in vivo *[47]. The previously mentioned regulator FNR and FNR-dependent small RNAs appear to be implicated in oxygen-dependent SOD abundance changes [48]. SodA and SodC were decreased *in vivo*, while iron-dependent SodB was clearly increased *in vivo*. An increase was noticed as well for the alkyl hydroperoxide reductase subunits AhpF/AhpC *in vivo*. The AhpC/AhpF subunits have also been implicated in the *S. typhimurium *defense in the macrophage environment [49]. The immune system in higher organisms including mice and humans generates reactive oxygen species, such as superoxide and peroxide ions to destroy invading microbes [50,51]. The increased abundance of oxidative stress proteins *in vivo *therefore implied a link to bacterial survival through evasion of the host immune response targeted against intestinal pathogens. Among the most dramatic abundance changes were those of three cold shock proteins (CspA, CspC and CspE). While the exact functions of these low M_r _proteins are not known, a recent study suggested that *csp *gene mutants reduced *Listeria monocytogenes *invasiveness [52], raising the question of their roles in epithelial or macrophage cell invasion by SD1. The dramatic abundance change of CspA (*in vivo vs. in vitro*) makes this protein a particularly interesting target for further characterization. Heat shock proteins (DnaK, IbpA, HtpG, GrpE) and chaperones (HslU/HslV, ClpB/ClpX) were also increased, indicative of further intracellular stress responses by the SD1 cells *in vivo*. In summary, we gained insight into protein expression changes likely required for the survival of *S. dysenteriae *during oxidative and acid stress in the host intestine.

### SD1 outer membrane and cell surface proteome

A large number of known or predicted β-barrel OM proteins were altered in abundance comparing *in vivo *and *in vitro *samples (OmpA, OmpC, IcsP/OmpT, OmpW/YciD, YaeT, Tsx, Lpp). Many of those proteins are either known or assumed to be exposed at the cell surface. A large number of lipoproteins sorted into the OM were also decreased in abundance under *in vivo *conditions (YoaF, LolB, SlyB, YcfM, NlpB, YfgL, NlpD). Proteins comprising the outer membrane YaeT protein assembly complex were decreased in abundance (YaeT, NlpB, YfgL) suggesting that the rate of biosynthesis and incorporation of the OM proteins was substantially decreased *in vivo*. Some of the chaperones presumably involved in OM protein transit and folding (YraP, HlpA, YtfJ), all part of RpoE regulon, were also decreased in abundance *in vivo *supporting the notion of reduced OM proteome turnover and a stress environment very different from that of stationary phase cells *in vitro*. Furthermore, components of the OM lipid asymmetry complex (YrbD, YrbF) and its regulator YrbA were decreased in abundance *in vivo*. This complex is a phospholipid transporter responsible for the balance of phospholipids and lipopolysaccharides in the outer leaflet of the OM. The outer membrane protein Imp directing the assembly of lipopolysaccharides was also decreased, while LolD, involved in translocation of OM lipoproteins, and the lipoprotein Nlpl (YhbM) were detected only *in vitro*. These changes, structurally or functionally associated with the OM, suggest a remodeling of the OM-associated cell surface, comprised of lipids, lipopolysaccharides and surface proteins, and a decreased turnover of proteins in the OM. Interestingly, the transcription factor BolA which regulates the transcription of genes involved in the morphology of the cell to confer cell protection was substantially decreased *in vivo*. Two other proteins likely involved in cell morphology and peptidoglycan turnover were also decreased in abundance under *in vivo *conditions, the rod-shape determining membrane protein YfgA and the LysM domain protein YgaU. It remains to be demonstrated whether these changes represent a coordinated physiological response of SD1 cells to the hostile environment in the host gut, possibly promoting evasion from the immune system and lowering OM porosity for protection from any extracellular toxic substances released by the host.

### *S. dysenteriae *type III secretion system and other virulence factors

The virulence plasmid encodes the 30 kb spa-mxi type III secretion system (TTSS) and invasion plasmid antigens (Ipa proteins) required for invasion of host cells [53]. The TTSS is comprised of a membrane-spanning protein complex which includes *ca*. 50 proteins, including Mxi and Spa proteins involved in assembly and regulation of the TTSS, chaperones (IpgA, IpgC, IpgE and Spa15), transcription activators (VirF, VirB and MxiE), translocators (IpaB, IpaC and IpaD) and *ca*. 25 effectors [8,54]. Invasion is followed by entry of *Shigella *into colonic epithelium cells *via *the basolateral membrane. Further bacterial invasion and lateral spreading of the bacteria within the colonic epithelium is mediated by host cell actin polymerization. The surface protein IcsA encoded by the virulence plasmid is responsible for actin-based motility required for intra- and inter-cellular spread of the bacteria [55]. *Shigella *manipulates the host innate and adaptive immune system *via *the Osp family of proteins [56].

In the present study, we identified many components of the TTSS, including 15 Mxi-Spa proteins and 16 effectors and their chaperones (Additional File 1, Table S1). The TTSS has been reported as being assembled with a few effectors and chaperones when cultured *in vitro*, and activated only after contact of bacteria with host cells [8]. Here, many TTSS proteins were identified in both the *in vitro *and *in vivo *datasets, including membrane associated Mxi and Spa proteins, Ipa effectors and Spa chaperones. Spa15 is a chaperone for the Osp family of effectors (OspC1, OspC2, OspC3) and also for the IpaA and IpgB2 effectors; while IpgC is a chaperone for IpaB and IpaC [8]. Activation of TTSS results in the induction of the transcription of genes encoding a second set of effectors under the control of MxiE and IpgC, including several *spa *genes. The OspC2 and OspC3 effectors and the IpgA and Spa32 proteins were detected only under *in vivo *conditions. Activation of the TTSS is followed by formation of the TTSS translocator pore which requires the IpaB, IpaC and IpaD effectors [5,57]. IpaB in particular induces apoptosis in host macrophages leading to inflammatory infection [58]. Several of the Ipa proteins (IpaA/B/C/D) were increased *in vivo*, while other TTSS effectors were either increased or detected solely *in vivo *(IpaJ, IpgD, IcsB, OspC2, OspC3, OspF, VirA). Additional plasmid-encoded proteins such as PhoN1 and PhoN2 were decreased in abundance *in vivo*. PhoN2 was reported to hydrolyze dNTPs and modulate the localization of IcsA at the bacterial cell surface [59]. OspC2, IpaB and VirB were identified as immunogenic when probed with a piglet antiserum in a 2D Western blot [15], suggesting that these proteins could form potential vaccine targets for the prevention of shigellosis. The Ipa proteins are known to be transiently associated with the cell surface and therefore are likely to contribute to the altered SD1 cell surface in the host gut environment. We assume that other proteins likely secreted *via *the TTSS (OspC2, OspC3) are at least transiently cell surface associated. Abundance changes of the TTSS virulence factors correlated well with the altered changes in the OM/cell surface proteins *in vivo*. We are tempted to speculate that the previously mentioned OM remodeling efforts benefit the adaptation of SD1 to the host cell invasion process *via *enhanced abundance of TTSS effectors in the cell envelope. However, our data do not support uniformly increased abundances of all detected TTSS proteins in the SD1 cell envelope *in vivo*.

The virulence of *Shigella *species is of the order *S. dysenteriae *>*S. flexneri *>*S. sonnei *>*S. boydii*. SD1 infection has a limited diarrheal phase with a sudden onset of acute dysentery, which could be explained by the expression of the potent virulence factor Shiga toxin (Stx) [14]. Shiga toxin subunit A (StxA) was detected only *in vitro*, while Shiga toxin subunit B (StxB) was detected both *in vitro *and *in vivo*, with StxB increased in abundance *in vitro*. As Stx is a secretory protein [14], the abundance levels of this protein are not readily obvious from proteomic profiling of cell lysates. It is of interest to examine whether the *Shigella *T2SS secretes other virulence factors in addition to the Shiga toxin. T2SS subunits were of very low abundance in SD1 cells according to this survey.

Other proteins involved in *Shigella *pathogenicity are the O-antigens which are highly diverse with at least 46 observed serotypes [2]. The variability of the O-antigens has been brought into context with evasion of the host immune system [60]. The small SD1 plasmid-encoded galactosyltransferase RfpB involved in the O-antigen biosynthesis was detected only *in vivo*, while other enzymes such as RfaD were increased *in vivo*. Enzymes potentially known to contribute monosaccharides (galactose and rhamnose) to the biosynthesis of the O-antigen sugars were also increased *in vivo*, including LacZ, GalE/K/M/T, RfbC, MelA, ManA and KdsB. Further studies are necessary to determine whether increased carbohydrate metabolism is functionally coupled to altered biosynthesis of O-antigen sugars under *in vivo *conditions.

## Conclusions

The comparative global proteomic survey of *S. dysenteriae *strain Sd1617 grown *in vitro vs. S. dysenteriae *cells isolated from an infected host animal model (*in vivo*) revealed abundance increases of several TTSS proteins and effectors under *in vivo *conditions. Virulence proteins such as OspC2 and IpaB, increased in abundance *in vivo*, were previously determined to be immunogenic, indicating their potential as vaccine candidates to combat shigellosis. Proteins important for the structural integrity of the bacterial cell wall and outer membrane such as OM proteins, lipoproteins, and chaperones for the cell envelope structures were decreased *in vivo*, indicating morphological changes in the bacterial cell wall. This hypothesis needs to be explored further in the context of infection, pathogenicity and protection from host factors. Proteins involved in response to anaerobic and nutrient deficient conditions, oxidative stress and acid stress were increased *in vivo*, reflecting the importance of the biochemical processes permitting the survival of the pathogen in the complex host gut environment. Further characterization of proteins increased in abundance *in vivo *will contribute to the understanding of host-pathogen interactions and facilitate the design of new vaccine candidates. It remains to be determined how the absence of microflora in the intestinal milieu might impact these observations.

## Abbreviations

APEX: absolute protein expression; CM: cytoplasmic membrane; LPS: lipopolysaccharide; MS: mass spectrometry; OM: outer membrane; T3SS: type III secretion system; TCA: tricarboxylic acid; TMD: transmembrane domain.

## Competing interests

The authors declare that they have no competing interests.

## Authors' contributions

SK - project conception and implementation, sample prep, generation of 2D-LC-MS/MS datasets and quantitation using the APEX Quantitative Proteomics Tool, bioinformatic, statistical and biological analyses of 2D-LC-MS/MS-APEX datasets, primary manuscript author, QZ - provided bacterial samples, manuscript author, JCB - software engineering and development of the APEX Quantitative Proteomics Tool, statistical and pathway analysis of APEX datasets, manuscript review, AD - project oversight, provided bacterial samples, manuscript review, ST - project oversight, provided bacterial samples, manuscript review, RP - project conception and implementation, participation in data interpretation and writing of the manuscript.

All authors read and approved the final manuscript.

## Supplementary Material

Additional file 1 **Table S1**. Protein abundance estimates from APEX quantitation. APEX abundance values of 1761 *S. dysenteriae *serotype 1 (SD1) *in vitro *and *in vivo *proteins quantitated at a <5% false discovery rate using the APEX Quantitative Proteomics Tool are listed along with their *pi*, *ni*, and *Oi *values. The corresponding gene names, locus tags, physicochemical properties and subcellular localizations are also listed in the table. Click here for file

Additional file 2 **Table S2**. SD1 differential protein expression statistical analysis using Z-test and SAM. SD1 proteins listed in blue are upregulated under *in vitro *conditions. For the two tailed Z-test, SD1 proteins differentially expressed at 99% confidence are listed; for the two class SAM test, proteins differentially expressed at <10% FDR are listed. Click here for file

Additional file 3 **Table S3**. APEX abundance values of SD1 *in vitro *and *in vivo *biological replicates. Three biological replicates from both *in vitro *and *in vivo *SD1 groups were analyzed as three to five technical replicates to expand the scope of the analysis; their APEX abundance values are listed. Click here for file
